# Use of Parenteral Antibiotics in Emergency Departments: Practice Patterns and Class Concordance

**DOI:** 10.5811/westjem.17998

**Published:** 2024-09-06

**Authors:** Megan Elli, Timothy Molinarolo, Aidan Mullan, Laura Walker

**Affiliations:** * Mayo Clinic, Department of Emergency Medicine, Rochester, Minnesota; † Mayo Clinic, Department of Pharmacy, Rochester, Minnesota; ‡ Mayo Clinic, Department of Quantitative Health Sciences, Rochester, Minnesota

## Abstract

**Introduction:**

We aimed to assess antibiotic stewardship by quantifying the use of first-dose intravenous (IV) vs oral-only antibiotics and the frequency with which antibiotic class was changed for discharged patients. Secondary aims included the following: evaluation of the relative length of stay (LOS); differences in prescribing patterns between clinician types; differences between academic and community settings; assessment of prescribing patterns among emergency department (ED) diagnoses; and frequency of return visits for patients in each group.

**Methods:**

This was a retrospective cohort study including patients presenting to EDs with infections who were discharged from our Midwest healthcare system consisting of 17 community hospitals and one academic center. We included infection type, antibiotic class and route of administration, type of infection, LOS, return visit within two weeks, clinician type, and demographics. Data were collected between June 1, 2018–December 31, 2021 and analyzed using descriptive statistics.

**Results:**

We had 77,204 ED visits for patients with infections during the study period, of whom 3,812 received IV antibiotics during their visit. There were more women (62.4%) than men included. Of the 3,812 patients who received IV antibiotics, 1,026 (34.3%) were discharged on a different class of antibiotics than they received. The most common changes were from IV cephalosporin to oral quinolone or penicillin. Patients treated with IV antibiotics prior to discharge had a longer LOS in the ED (median difference of 102 minutes longer for those who received IV antibiotics). There was not a significant difference in the use of IV antibiotics between the academic center and community sites included in the study.

**Conclusion:**

Administering IV antibiotics as a first dose prior to oral prescriptions upon discharge is common, as is shifting classes from the IV dose to the oral prescription. This offers an opportunity for intervention to improve antibiotic stewardship for ED patients as well as reduce cost and length of stay.

Population Health Research Capsule What do we already know about this issue?

*Antibiotic stewardship is imperative due to rising rates of antibiotic-related infections and resistance; it is crucial that antibiotics be prescribed appropriately.*

What was the research question?

*We quantified the use of first-dose IV vs oral-only antibiotics and the frequency that antibiotic class was changed.*

What was the major finding of the study?

*Of 3,812 patients (4.9%, 95% CI 4.8–5.1%) who received IV antibiotics in the ED, 1,273 (33.4%, 95% CI 31.9–34.9%) were prescribed a different antibiotic class when discharged.*

How does this improve population health?

*By recognizing inconsistencies in patients treated with antibiotics in the ED prior to discharge, this presents future opportunities to improve upon antibiotic stewardship.*

## INTRODUCTION

### Background

Acute infections are a common reason for patients to present to the emergency department (ED). There were approximately 130 million ED visits in 2018 within the United States. Acute infections account for approximately 15 million visits to the ED annually.^1^ ^,^ ^2^ Acute respiratory infections, skin infections (eg, cellulitis and subcutaneous abscess), and urinary tract infections (UTI) are among the most common infections evaluated in the ED in recent years.^3^

Prior work has addressed inpatient concerns about transitioning from intravenous (IV) to oral antibiotics, some of which are applicable to the ED setting. These include concerns for no clear benefit; reduced bioavailability; inapplicability of bioavailability studies performed on healthy individuals; improved clinical outcomes with IV therapy; and concern that an oral route of administration may contribute to an infection lingering. These concerns are systematically addressed and include commentary on the bioavailability of most antibiotics, which is greater than 90%. Many of the classes can achieve a serum concentration that is the same via oral or IV routes. Treatment with IV antibiotics compared with oral antibiotics has been noted to be more expensive than oral antibiotics alone, and IV antibiotics also have the risk of possible complications associated with IV-line insertion and use.^4^ Cephalosporins are a notable exception and have been studied in the setting of pyelonephritis with recommendations for a single IV dose in the ED prior to dismissal (Gupta et al, below). However, the serum concentration achievable via oral administration is adequate to treat mild and moderate infections,^5^ as we would expect to see in a patient who is discharged home.

### Importance

Antibiotic stewardship is increasingly important due to the rising rates of antibiotic resistance including methicillin-resistant *Staphylococcus aureus* and multidrug resistant bacteria in the setting of pneumonia and UTIs.^6^ ^–^ ^8^ There are also risks of antibiotic-related complications such as *Clostridioides difficile* colitis. It is, therefore, imperative that antibiotics be administered for the shortest duration required, through an appropriate route, and prescribed only when necessary.^9^ There is scant information regarding the concordance between first dose of IV antibiotics and subsequent prescription from the ED. There are differences in the financial burden and time associated with the administration route of antibiotics. Oral antibiotics are more cost effective than IV antibiotics and often provide similar microbial coverage.^4^ Multiple studies have compared oral and IV antibiotics when treating UTIs, cellulitis, or pneumonia separately, but studies are lacking that evaluate oral and IV antibiotics for treating infections in a broader sense.^10^ ^–^ ^12^ In many cases, oral antibiotics may be appropriate and as efficacious as IV antibiotics, providing more time-efficient and cost-effective care for patients who are discharged from the ED.

### Goals of This Investigation

Our primary aim was to determine whether we adhere to best practices in prescribing antibiotics for common infectious conditions being treated on an outpatient basis after an ED visit. This includes the route of administration and concordance between any doses given in the ED and subsequent prescriptions. Secondary aims included the following: evaluation of the relative length of stay (LOS) for patients receiving a dose of IV antibiotics who are subsequently discharged compared to those given oral antibiotics only; differences in prescribing patterns between clinician types (physician, physician assistant/nurse practitioner) and in academic vs community settings; assessment of prescribing patterns among ED diagnoses (eg, skin/soft tissue, urinary, pulmonary); and patterns of return visits for patients receiving a dose of parenteral vs oral antibiotics only.

## METHODS

### Study Design and Setting

This study adheres to the STROBE guidelines for reporting observational studies.^13^ This was a multicenter retrospective cohort study. We included patients in a single academic center and 17 community EDs affiliated with our institution located throughout the Midwest.

### Selection of Participants

We included patients who were evaluated and discharged from the ED with a diagnosis of infection, based on International Classification of Diseases (ICD-10) codes, and were prescribed oral antibiotics upon discharge from the ED. Some patients received oral antibiotics following a dose of IV antibiotics provided prior to discharge. Patients who were admitted to the hospital or placed in ED observation during their first ED visit were excluded from our analysis.

### Measurements

Our dataset included the following: visit identifier; legal gender; gender identify; primary language; age at visit; antibiotics given in ED (yes/no); allergies; ED location; ED arrival time; ED departure time; chief complaint; primary diagnosis; diagnosis list; final disposition; first attending, last attending, resident, advanced practice practitioner (APP), primary nurse; return visit identifier; return visit location; days between return visits; return visit arrival time; return visit departure time; return visit chief complaint; return visit primary diagnosis; return visit disposition; antibiotic order identification; outpatient antibiotic order date; outpatient antibiotic prescription; and outpatient antibiotic prescriber and specialty.

We categorized antibiotics by their route of administration (parenteral vs oral) and by pharmacologic class (aminoglycoside, carbapenem, cephalosporin, epoxide, glycopeptide, lincomycin, macrolide, nitrofurantoin, nitroimidazole, penicillin, quinolone, sulfa, or tetracycline). Topical is included among the classes of antibiotics due to its distinct use. Antibiotics were considered concordant if the antibiotic provided parenterally was in the same class as the oral antibiotic prescribed upon discharge. Prescribing patterns were evaluated based on credentials with subgroups of physicians (MD/DO/MBBS) and advanced practice providers (nurse practitioner [NP]/physician assistant [PA]). Practice settings were defined as an academic center that includes an emergency medicine residency program and community-based settings. The ICD-10 diagnoses were grouped based on organ system with presumed bacterial etiology (urinary, skin/soft tissue, pulmonary, gastrointestinal, otolaryngological, animal bite, insect bite, dental, orthopedic, ophthalmologic, osteomyelitis) and/or organism type (fungal and parasitic) and categories for fever of unknown origin, postoperative infections, prophylaxis, and other infections, which is a catch-all for uncommon diagnoses. A complete list of the ICD-10 associated diagnoses included within the study is available in Appendix A. Length of stay (LOS) is measured in time elapsed from presentation to the ED until the time of discharge. Return visits were considered potentially related to the index visit for infection if they occurred within two weeks.

### Outcomes

The primary outcome was the route of administration of antibiotics for a diagnosed infection and concordance of prescription oral antibiotics with any parenteral treatment given. Secondary outcomes included LOS within the ED, differences in prescribing IV or oral antibiotics between physicians and APPs, differences between academic and community setting, and association between treatment and ED return visits.

### Analysis

We summarized continuous features were summarized with means with standard deviations, as well as medians with interquartile ranges; categorical features were summarized with frequency counts and percentages. We calculated confidence intervals (CI) for percentages using an exact binomial distribution. Demographics and visit characteristics were compared between patients who received IV antibiotics and patients who did not, using Wilcoxon rank-sum tests and chi-squared tests.

For the main outcomes of interest, we compared the rates of treatment with IV antibiotics by ED practice and clinician type using chi-squared tests. Similarly, the rate of two-week ED returns was compared between patients treated with both IV and prescription antibiotics and patients treated only with prescription antibiotics, using chi-squared tests. We compared ED LOS between patients receiving IV antibiotics in the ED and patients not treated with IV antibiotics, using Wilcoxon rank-sum tests. Test results were reported with the median difference in LOS times along with 95% CIs calculated by bootstrap resampling. All tests were two-sided and *P*-values less than 0.05 were considered significant. Analysis was performed using R version 4.1.2 (R Foundation for Statistical Computing, Vienna, Austria).^14^

## RESULTS

### Characteristics of Study Subjects

Patient demographics and visit characteristics are summarized in Table 1. Of note, there were significantly more female than male subjects (62.4% vs 37.6%). The median age of patients was different between patients who did and did not receive IV antibiotics. There was a median age of 50.0 years for those who did not receive IV antibiotics compared to a median age of 55.5 years for those who did. The majority of the patients included within the study were English speaking (97.2%).

**Table 1.** Demographics and visit characteristics.

	No IV Abx in the ED (N = 73,392)	IV Abx in the ED (N = 3,812)	*P*-value
Patient gender			< 0.001
Female (n = 48,157)	45,600 (62.1%)	2,557 (67.1%)	
Male (n = 29,045)	27,790 (37.9%)	1,255 (32.9%)	
Unknown	2 (0.0%)	0 (0.0%)	
Patient age			< 0.001
Mean (SD)	49.2 (24.5)	53.4 (23.6)	
Median (Q1, Q3)	50.0 (29.0, 69.0)	55.5 (33.0, 73.0)	
Primary language			< 0.001
English	71,389 (97.3%)	3,686 (96.7%)	
Non-English	1,918 (2.6%)	121 (3.2%)	
Unknown/did not disclose	85 (0.1%)	5 (0.1%)	
ED practice type			0.14
Academic center	22,591 (30.8%)	1,130 (29.6%)	
Community practice	50,801 (69.2%)	2,682 (70.4%)	
Clinician type			< 0.001
NP/PA	28,976 (39.5%)	1,231 (32.3%)	
Physician	44,314 (60.5%)	2,578 (67.7%)	
ED length of stay			< 0.001
Mean (SD)	164.3 (128.8)	278.3 (183.6)	
Median (Q1, Q3)	139.0 (76.0, 219.0)	241.0 (180.0, 314.0)	
Change in antibiotic class			< 0.001
No IV antibiotics	73,392 (100.0%)	–	
Changed antibiotics	0 (0.0%)	1,273 (33.4%)	
Same antibiotics	0 (0.0%)	2,539 (66.6%)	

*IV*, intravenous; *Abx*, antibiotics; *ED*, emergency department; *Q1*, first quartile; *Q3*, third quartile; *NP/PA*, nurse practitioner or physician assistant.

### Main Results

A total of 77,204 ED visits for patients with infections occurred between June 1, 2018–December 31, 2021 among all sites and were included for analysis. There were 3,812 patients (4.9%, 95% CI 4.8–5.1%) who received IV antibiotics in the ED. Nearly all the patients who received IV antibiotics within the ED received cephalosporins (3,637 patients, 95.4%), with penicillins (114 patients, 3.0%) and glycopeptides (32 patients, 0.8%) being the next most common. The primary infectious diagnoses are summarized within Table 2. Patients diagnosed with a UTI were the largest group treated with IV antibiotics (63.3%). When comparing pyelonephritis with other UTIs, pyelonephritis was much more likely to be treated with IV antibiotics than all other UTIs (28.3% vs 6.5%, *P* < 0.001). Insect bites (0.2%) and bite wounds (33 of 4,047 visits, 0.8%) received IV antibiotics the least often.

**Table 2.** Summary of antibiotic, antifungal, antiparasitic treatment type for emergency department infections.

Primary ED diagnosis	IV Abx in the ED (N = 2,989)	No IV Abx in the ED (N = 56,289)	Percent of diagnosed with IV Abx in the ED
UTIs not diagnosed as pyelonephritis	1,145 (38.3%)	16,591 (29.5%)	6.5%
Pyelonephritis	747 (25.0%)	1,888 (3.35%)	28.3%
Skin/soft tissue infection	540 (18.1%)	20,109 (35.7%)	2.6%
Pulmonary infection	361 (12.1%)	6,708 (11.9%)	5.1%
Gastrointestinal infection	87 (2.9%)	4,211 (7.5%)	2.0%
ENT infection	36 (1.2%)	667 (1.2%)	5.1%
Bite wound	33 (1.1%)	4,014 (7.1%)	0.8%
FUO	15 (0.5%)	207 (0.4%)	6.8%
Other infection	10 (0.3%)	66 (0.1%)	13.2%
Dental infection	9 (0.3%)	454 (0.8%)	1.9%
Insect bite	2 (0.1%)	1,096 (1.9%)	0.2%
Orthopedic infection	2 (0.1%)	62 (0.1%)	3.1%
Ophthalmologic infection	1 (0.0%)	68 (0.1%)	1.4%
Fungal infection	1 (0.0%)	61 (0.1%)	1.6%
Prophylaxis	0 (0.0%)	52 (0.1%)	0%
Post-op infection	0 (0.0%)	22 (0.0%)	0%
Parasitic infection	0 (0.0%)	8 (0.0%)	0%
Osteomyelitis	0 (0.0%)	4 (0.0%)	0%

*IV*, intravenous; *Abx*, antibiotic/antifungal/antiparasitic; *ED*, emergency department; *UTI*, urinary tract infection; *ENT*, otolaryngological; *FUO*, fever of unknown origin.

Table 3 compares the IV antibiotic administered in the ED to oral antibiotic prescribed upon discharge. The most common changes in antibiotic class were from IV cephalosporin to oral quinolone (304 visits, 23.9% of changes) and from IV cephalosporin to oral penicillin (231 visits, 18.1% of changes). The most common oral antibiotic class prescribed at discharge from the ED was cephalosporin. The most commonly prescribed topical antibiotic was within the class of cephalosporins as well. The type of antibiotic prescribed to patients treated parenterally was different from the those prescribed for oral-only treatment (*P* < 0.001). Among the patients treated with IV antibiotics, 1,273 (33.4%, 95% CI 31.9–34.9%) received a prescription for a different antibiotic class at discharge.

**Table 3.** Comparison of intravenous and prescription antibiotics, antifungals, and antiparasitic agents.

		IV antibiotic class administered in the ED							
		Amino-glycoside (N = 2)	Carba-penem (N = 6)	Cephalo-sporin (N = 3,637)	Glyco-peptide (N = 32)	Lincomycin (N = 1)	PCN (N = 114)	Sulfa (N = 1)	Tetra-cycline (N = 19)
Oral antibiotic class prescribed at discharge	Aminoglycoside	0 (0%)	0 (0%)	1 (0.0%)	0 (0%)	0 (0%)	0 (0%)	0 (0%)	0 (0%)
	Carbapenem	0 (0%)	3 (50%)	0 (0%)	0 (0%)	0 (0%)	0 (0%)	0 (0%)	0 (0%)
	Cephalosporin	0 (0%)	0 (0%)	2,431 (66.8%)	6 (19%)	0 (0%)	4 (3.5%)	0 (0%)	3 (16%)
	Epoxide	0 (0%)	1 (17%)	1 (0.0%)	0 (0%)	0 (0%)	0 (0%)	0 (0%)	0 (0%)
	Glycopeptide	0 (0%)	0 (0%)	2 (0.1%)	0 (0%)	0 (0%)	0 (0%)	0 (0%)	0 (0%)
	Lincomycin	0 (0%)	0 (0%)	25 (0.7%)	6 (19%)	1 (100%)	5 (4.4%)	0 (0%)	0 (0%)
	Macrolide	0 (0%)	0 (0%)	172 (4.7%)	0 (0%)	0 (0%)	4 (3.5%)	0 (0%)	0 (0%)
	Nitrofuran	1 (50%)	0 (0%)	86 (2.4%)	0 (0%)	0 (0%)	0 (0%)	0 (0%)	0 (0%)
	Nitroimidazole	0 (0%)	0 (0%)	50 (1.4%)	0 (0%)	0 (0%)	1 (0.9%)	0 (0%)	1 (5.3%)
	PCN	0 (0%)	0 (0%)	231 (6.4%)	2 (6%)	0 (0%)	88 (77.2%)	0 (0%)	0 (0%)
	Quinolone	0 (0%)	2 (33%)	304 (8.4%)	3 (9%)	0 (0%)	5 (1.8%)	0 (0%)	0 (0%)
	Sulfa	1 (50%)	0 (0%)	179 (4.9%)	8 (25%)	0 (0%)	2 (1.8%)	1 (100%)	0 (0%)
	Tetracycline	0 (0%)	0 (0%)	153 (4.2%)	6 (19%)	0 (0%)	4 (3.5%)	0 (0%)	15 (79%)
	Topical	0 (0%)	0 (0%)	2 (0.1%)	1 (3%)	0 (0%)	1 (0.9%)	0 (0%)	0 (0%)

Shaded cells indicate concordant intravenous and prescription antibiotic class.

*IV*, intravenous; *ED*, emergency department; *PCN*, penicillin.

#### Secondary outcomes

For our secondary end points, we found that patients treated with IV antibiotics prior to discharge had a longer LOS within the ED (median difference 102 minutes longer for those who received IV antibiotics; 95% CI 97–106 minutes; *P* < 0.001). Physicians were more likely to treat patients with IV antibiotics compared to APPS (5.5% vs 4.1%; *P* < 0.001). While this is statistically significant, the overall percentage difference is small. There was no significant difference in the use of IV antibiotics between the academic center and community sites (4.8% vs 5.0%; *P* = 0.14).

When we assessed diagnosis-based patterns, we found that among patients given IV antibiotics during the ED visit, the group most likely to be prescribed a different class of antibiotic was those with pulmonary infections (279 of 361 visits, 77.3%) followed by gastrointestinal (54 of 87 visits, 62.1%). There was one patient with an ophthalmologic infection, and the class of antibiotics was changed upon dismissal (Table 4). Patients treated with IV antibiotics for UTIs were least likely to change antibiotic class at dismissal (20.7% of 391 visits). The IV and oral antibiotics class administered based on the infection type is summarized within Table 5. Among the 3,812 patients who received parenteral antibiotics during their ED visit, 749 (19.6%), had a return visit within two weeks compared to 11,601 (15.8%) of 73,392 patients who received only oral antibiotics (*P* < 0.001).

**Table 4.** Change in antibiotics and antifungals by infection diagnosis.

Primary diagnosis	Changed antibiotics (N = 1,026)		Same antibiotics (N = 1,963)
Ophthalmologic infection	1 (100%)		0 (0.0%)
Pulmonary infection	279 (77.3%)		82 (22.7%)
Gastrointestinal infection	54 (62.1%)		33 (37.9%)
ENT infection	21 (58.3%)		15 (41.7%)
Insect bite	1 (50.0%)		1 (50.0%)
Other infection	5 (50.0%)		5 (50.0%)
Orthopedic infection	1 (50.0%)		1 (50.0%)
Skin/soft tissue infection	256 (47.4%)		284 (52.6%)
Dental infection	4 (44.4%)		5 (55.6%)
FUO	6 (40.0%)		9 (60.0%)
Bite wound	7 (21.2%)		26 (78.8%)
Urinary infection	391 (20.7%)		1,501 (79.3%)
Fungal infection	0 (0.0%)		1 (100%)
Prophylaxis	0 (0.0%)		0 (0.0%)
Post-op infection	0 (0.0%)		0 (0.0%)
Parasitic infection	0 (0.0%)		0 (0.0%)
Osteomyelitis	0 (0.0%)		0 (0.0%)

1 Percentages are calculated row-wise, relative to the total number of patients within each primary diagnosis group.

*ENT*, otolaryngological; *FUO*, fever of unknown origin.

**Table 5.** Intravenous antibiotics and antifungals administered by infection type.

	IV antibiotics in the ED				
Primary diagnosis	Cephalosporin (N = 2,830)	PCN (N = 103)	Glycopeptide (N = 29)	Tetracycline (N = 17)	Other Abx (N = 10)
FUO	15 (100%)	0 (0%)	0 (0%)	0 (0%)	0 (0%)
Other infection	10 (100%)	0 (0%)	0 (0%)	0 (0%)	0 (0%)
Orthopedic infection	2 (100%)	0 (0%)	0 (0%)	0 (0%)	0 (0%)
Insect bite	2 (100%)	0 (0.0%)	0 (0%)	0 (0%)	0 (0%)
Fungal infection	1 (100%)	0 (0%)	0 (0%)	0 (0%)	0 (0%)
Urinary infection	1,879 (99.3%)	4 (0.2%)	2 (0.1%)	0 (0%)	7 (0.4%)
Pulmonary infection	354 (98.1%)	5 (1.4%)	0 (0%)	2 (0.6%)	0 (0%)
GI infection	74 (85.1%)	12 (13.8%)	0 (0%)	0 (0%)	1 (1.1%)
Skin/soft tissue Infection	462 (82.6%)	38 (7.0%)	26 (4.8%)	13 (2.4%)	1 (0.2%)
ENT infection	22 (61.1%)	13 (36.1%)	0 (0%)	0 (0%)	1 (2.8%)
Dental infection	2 (22.2%)	6 (66.7%)	1 (11.1%)	0 (0%)	0 (0%)
Bite wound	7 (21.2%)	24 (72.7%)	0 (0%)	2 (6.1%)	0 (0%)
Ophthalmologic infection	0 (0%)	1 (100%)	0 (0%)	0 (0%)	0 (0%)

1 Percentages are calculated row-wise, relative to the total number of patients within each primary diagnosis group.

*IV*, intravenous; *ED*, emergency department; *GI*, gastrointestinal; *ENT*, otolaryngological; *FUO*, fever of unknown origin; *Abx*, antibiotics.

## DISCUSSION

Our analysis of ED visits by patients with common infectious diseases who were treated with antibiotics revealed that there are opportunities for improvement in selection of antibiotics in terms of administration route and home-going prescriptions in our hospital system. Recommendations from the Infectious Disease Society of America (IDSA) are available for the three most common areas of infection among our patients: urinary; skin; and pulmonary sources. The IDSA guidelines regarding treatment for UTIs recommend oral treatment for uncomplicated cystitis, and while oral antibiotics are also appropriate for acute pyelonephritis, there is an option to provide a one-time IV dose of antibiotics, such as a long-acting cephalosporin, prior to initiation of oral therapy.^15^ For skin infections, the IDSA guidelines use a mild, moderate, and severe grading for cellulitis. Only mild is categorized as appropriate for oral therapy; moderate and severe are recommended to receive IV antibiotics. There are multiple appropriate oral and IV options for treatment of bite wounds.^16^ First-line treatment options for outpatient community-acquired pneumonia include oral amoxicillin, macrolides, and doxycycline for patients with few risk factors, and amoxicillin-clavulanate in conjunction with atypical coverage.^17^ Healthcare-associated pneumonia treatment recommendations often include multiple medications typically including a required IV agent, such as vancomycin, precluding discharge.^18^

Patients who present to the ED for care are often complex; clinical assessment of multiple factors including clinical gestalt, in addition to laboratory and imaging findings, may cue a clinician to have a higher suspicion for a severe infection, thus prompting them to provide IV treatment. Additionally, there could be some diagnostic uncertainty prompting a desire to initiate empiric treatment prior to attaining a definitive diagnosis. Patients often improve while under our care, and it is possible that a patient is expected to be admitted to the hospital and provided IV antibiotics and either improves enough for dismissal, or perhaps they do not want to be admitted. The number of scenarios is nearly limitless. There is no clear answer as to how decisions are made to deviate from recommendations, and it may be an area ripe for additional research to understand the basis.

We identified that for UTIs, we had the highest concordance rate when an IV dose of antibiotics is prescribed. This is an opportunity to explore the relative cost of IV vs oral therapies. Using drugs.com, we found that an IV dose of 1 gram ceftriaxone costs approximately $11.47, prior to reconstitution. A dose of oral cefdinir costs under $2. In addition to the cost of the medications, there are additional charges associated with IV catheter placement and medication administration.^19^

Use of a single-dose glycopeptide (vancomycin) is one of the more problematic examples within our study. Unsurprisingly, we found that there were no episodes of concordance between IV and oral administration for vancomycin. The cost for vancomycin is approximately $200 for IV solution.^19^ In addition to the cost for the medication and administration, the cost of time increases with vancomycin, given its longer administration time compared to other IV antibiotics or oral-only therapy.

Our study showed that there is a significant difference in LOS, which impacts ED throughput and crowding, as well as patient quality of life. We cannot attribute this difference solely to the provision of IV antibiotics, and it may be due to other confounding factors. However, in a time in which ED crowding and prompt throughput is a matter of patient safety, it should not be neglected. There is an additional cost to the institution for the occupancy of a bed in the ED. Schreyer et al calculated the personnel cost for a single bed-hour in the ED to be $58.20.^20^ While over 3,000 patients who received IV antibiotics have an average LOS of 100 minutes greater than the patients treated with oral antibiotics, we find a substantial financial impact in addition to a quality-of-care effect.^20^

We found no difference in prescribing patterns between community and academic settings and only a small difference between physician and APPs. This may reflect practice patterns established by institutional norms, training programs that perpetuate a similar culture being passed on from supervisor to trainee, or simply common practices in emergency medicine.

The final outcome we examined was the likelihood to have a second visit within two weeks and whether there was a difference in the IV-oral vs oral-only groups (19.6% vs 15.8%). We were surprised to find that the patients who received IV antibiotics were more likely to return. This could have been related to discordance between IV antibiotics administered within the ED and oral antibiotics that patients received upon discharge. Or it may reflect a more severe disease than was appreciated by the treating team, resulting in the administration of IV antibiotics, or patients who declined admission. Further investigation into the course of these patients may shed additional light on the clinical decision-making around medication administration, prescription, and anticipated trajectory of their illness.

An additional finding that we discovered was the predominance of women as recipients of IV antibiotics. This is consistent with the higher incidence of UTIs in women as compared to men,^21^ which in combination with the high numbers of patients who received IV antibiotics with UTI/pyelonephritis could account for this finding. Given the higher cost of care due to IV medication administration and longer duration of time spent in the ED, it is important to consider the disparities in downstream effects of treatment between genders.

### Future Opportunities

Opportunities for further research include investigating any variation in average duration of illness, cost of care, or patient satisfaction between patients who receive oral antibiotics alone compared with patients who initially receive IV antibiotics. Evaluating the reasons for administering IV antibiotics initially and the reason for changing from one class of IV antibiotics to another class of oral antibiotics in the ED setting is worth further inquiry as well. Identifying the underlying cause for prescribing behaviors that are not adherent to recommended best practices will reveal opportunities for education and intervention. Providing education regarding oral bioavailability and efficacy of appropriate antibiotics may be helpful. These may include education on pharmacokinetics, implementation of electronic health record decision support, processes for prescriber and pharmacist collaboration, and more. Additionally, clarification of the IDSA guidelines around first-line treatment may result in improved LOS in the ED and other patient-oriented outcomes. In particular, the use of an IV dose of vancomycin prior to dismissal on other agents is a prime area for intervention with its associated costs and duration of administration.

## LIMITATIONS

This was a retrospective cohort study, which has the associated limitations related to bias. Our study sample was found to be skewed toward female gender compared to the general population. Additionally, the large number of primary English-speaking patients may be an indicator that this study is not generalizable to EDs in more diverse settings. Our data was not able to detect the clinical significance related to the return visits. More patients who received IV antibiotics returned to the ED, but it is not clear whether this was related to the underlying infection, whether IV antibiotics were prescribed due to a clinical judgment that the patient appeared more ill and was at higher risk of disease progression, or whether other factors influenced this trajectory. No surrogates for patient acuity were included in our analysis. Inclusion of an illness severity score could improve the ability to understand the decision to provide parenteral antibiotics, as well as inform the context regarding return visits and provide additional understanding of the difference in LOS. When comparing prescribing differences between physicians and APPs we did not control for practice setting, which ranges from a NP/PA with independent practice at a critical access hospital or within an academic ED and may or may not include direct on-site supervision. Neither did we control for the presence of an ED-based pharmacist to assist with prescribing recommendations.

## CONCLUSION

We found that patients within our analysis who were treated with intravenous antibiotics in the ED often received a different class of oral antibiotics upon discharge. We also found that administering IV antibiotics as a first dose prior to an oral antibiotic being prescribed upon discharge from the ED was common but may not be necessary. By recognizing these inconsistencies, there are future opportunities to improve upon antibiotic stewardship and adherence for prescribing oral antibiotics that are concordant with IV antibiotics that are administered.

## Supplementary Information




